# The impact of betamethasone on fetal pulmonary, umbilical and middle cerebral artery Doppler velocimetry and its relationship with neonatal respiratory distress syndrome

**DOI:** 10.1186/s12884-021-03655-2

**Published:** 2021-03-06

**Authors:** Homeira Vafaei, Fahimeh Kaveh Baghbahadorani, Nasrin Asadi, Maryam Kasraeian, Azam Faraji, Shohreh Roozmeh, Marjan Zare, Khadije Bazrafshan

**Affiliations:** 1 Maternal-Fetal Medicine Research Center, Shiraz University of Medical Sciences, Shiraz, Iran; 2 Maternal-Fetal Medicine Research Center, Perinatology Department, Shiraz University of Medical Sciences, Shiraz, Iran

**Keywords:** Fetal pulmonary artery, Fetal lung maturity, Betamethasone, Respiratory distress syndrome

## Abstract

**Background:**

Prenatal corticosteroid administration is known to be an effective strategy in improving fetal pulmonary maturity. This study aimed to evaluate the impact of maternal betamethasone administration on fetal pulmonary and other arteries Doppler velocity and the correlation between RDS development and Doppler indices results.

**Methods:**

Fifty one singleton pregnancies between 26 and 34 gestational weeks with a diagnosis of preterm labor were included in the exposed group and received betamethasone. Fifty one uncomplicated pregnancies were included in the non-exposed group. Fetal pulmonary, umbilical and middle cerebral arteries Doppler parameters were evaluated before and 24 to 48 h after steroid administration in the exposed group and two times at same intervals in the non-exposed group. Maternal records were matched to neonatal charts if delivery happened, and demographic and outcome data were abstracted.

**Results:**

When compared with the nonexposed group, fetuses treated with corticosteroids demonstrated significantly decreased umbilical artery Pulsatility index (PI) and significantly increased the middle cerebral artery PI, pulmonary artery Acceleration time (AT) and pulmonary artery AT/ET (Ejection time), while all other indices remained similar. We found significantly decreased pulmonary artery AT in the fetuses with respiratory distress syndrome (RDS) compared to those that did not.

**Conclusions:**

The results of our study showed that maternal antenatal betamethasone administration caused significant changes in the fetus blood velocity waveforms and also affected the blood flow in the pulmonary artery which led to an increase in the pulmonary artery AT and AT/ET. Among those fetuses with RDS, we found a significant decrease in the pulmonary artery AT, but we did not observe any pulmonary artery AT/ET differences.

## Introduction

Preterm birth, described by the world health organization (WHO) as delivery before 37 weeks of gestation, is a leading cause of neonatal morbidity and mortality [1].

Preterm birth is a risk factor in over 50 % of all neonatal deaths. In addition, preterm birth can result in a range of problems and long-term loss of human potential among survivors. Respiratory distress syndrome due to immaturity of fetal lungs, is one of the major complications for the fetus in preterm labor [1, 2].

The past few decades have seen significant development in outcomes for preterm infants due to the introduction of sophisticated neonatal ventilation, the use of exogenous surfactant, and the administration of antenatal steroid. When used appropriately, antenatal steroid administration is one of the most important and beneficial treatments used in perinatal medicine today. The two glucocorticoids in most common clinical use are dexamethasone and betamethasone [2, 3]. There are two different courses of standard corticosteroid therapy to accelerate fetal lung maturation: 1. Administration of betamethasone 12 mg intramuscularly and repetition of the same dose 24 h later 2. Dexamethasone 6 mg intravenously and its repetition every 12 h for up to 4 doses [4, 5]. The mechanism by which corticosteroids improve the respiratory status is unclear.

Antenatal administration of corticosteroids accelerate lung maturation. The normal thinning of the double capillary loops, to form the thin gas exchanging walls of alveoli, is accelerated, resulting in fast alveolisation. The maturation of surfactant producing type II pneumocytes is also speeded up. Although the alveolisation occurs rapidly as a result of the corticosteroids [2, 6].

Pulmonary surfactant is produced by type II pneumocyte. Surfactant phospholipids have surface tension reducing properties, while the surfactant proteins are important in regulating surfactant function, and may also have an immunomodulatory role [6, 7].

Evaluation of fetal lung maturity is one of the critical issues in managing pregnant patients at risk of preterm delivery. Diagnosis of fetal lung maturity is traditionally based on amniocentesis and measurement of the lecithin to sphingomyelin ratio, phosphatidylglycerol, fluorescence polarization test, foam or shake test, and counting the number of lamellar bodies in the amniotic fluid. Nevertheless, amniocentesis is an invasive procedure that must be performed according to its indications [8–10].

For these reasons, the use of noninvasive methods such as ultrasound to assess the fetal lung maturity has been considered in various cases. So far, efforts to predict fetal lung maturity based on ultrasound examination of lung volume, epiphysis centers, placental grading, and estimated fetal weight have been performed but has not been very successful in the clinical application [11–14].

During pregnancy, as the fetal lung develops, the vascular structure of the lungs changes, the amount of the smooth muscle tissue increases, and the level of pulmonary vascular resistance decreases, leading to a gradual increase in pulmonary blood flow. Doppler velocimetry can be a noninvasive method for assessing the fetal lung blood flow [15, 16].

Prenatal corticosteroid administration may increase the production of surfactant in the fetal lungs, which may increase the pulmonary artery blood flow probably due to accelerated lung maturation and its effect on the vascular structure [15, 17, 18] .

Although effects of betamethasone on fetal hemodynamics have been documented in some studies, the possible mechanisms underlying these alterations are still unclear. One of the possible theories to explain modifications in the fetal circulation associated with reduced placental resistance is based on an increased secretion of placental corticotropin-releasing hormone (CRH) after exogenous administration of corticosteroids, which consecutively causes nitric oxide-mediated vasodilatation [19, 20].

Another possibility is associated with increased fetal blood pressure. Experimental studies have shown that administration of betamethasone to fetal sheep causes an increase in blood pressure [21]. Furthermore, antenatal corticosteroid administration has been found to increase blood pressure levels in preterm newborns during the first days of life [22] .

This study aimed to evaluate the effect of maternal betamethasone administration on fetal pulmonary, umbilical and middle cerebral arteries Doppler indices and to investigate their relationship with the incidence of neonatal respiratory distress syndrome.

## Materials and methods

We conducted a prospective cohort study between October 2019 and August 2020. This study was approved by the ethics committee of Shiraz University of Medical Sciences (ethics code: IR.SUMS.REC.1399.351) and written informed consent was obtained from all participants in the study. In the exposed group, singleton pregnancies between 26 and 34 gestational weeks with a diagnosis of preterm labor (evidence of cervical dilatation or uterine contraction in nonstress test) who were candidates for corticosteroid administration for lung maturity and had the inclusion criteria were included. In the nonexposed group, uncomplicated singleton pregnancies in the same gestational age range, which had the inclusion criteria, were included.

The inclusion criteria in this study were: existence of appropriate evidence to determine the gestational age, gestational age between 26 and 34 weeks, no consumption of corticosteroids during pregnancy, estimated fetal weight between 10 and 90 percentile, average amniotic fluid volume. Also, lack of evidence of preeclampsia, diabetes, hypertension, peripheral vascular disease, underlying disease requiring corticosteroids, preterm premature rupture of membrane, and vaginal bleeding in the mother.

Exclusion criteria in this study included the existence of chromosomal or structural abnormality in the fetus and occurrence of labor before the second ultrasonography Doppler evaluation.

After reviewing the inclusion criteria, 63 patients with the diagnosis of preterm birth and indication for corticosteroid administration in the exposure group were included in the study. Besides, 54 healthy pregnant women without preterm labor symptoms referred for routine pregnancy care with similar demographic characteristics to the exposed group were included in the study as a nonexposed group by observing other inclusion criteria.

However, during the study, 12 subjects from the exposure group and 3 from the non-exposure group were excluded. Finally, the information of 51 people in each group were completed and used for the final analysis. The study population Fig. 2 shows the number of cases included, repeated measurements and the cases excluded.

After entering the study, information about each participant including the maternal age, demographic characteristics, and gestational age were recorded in the form for each patient. Gestational age was determined based on the patient’s last menstrual information and matched with the first trimester ultrasound.

This study was performed using a GE Voluson E6 (GE Healthcare) ultrasound machine equipped with a 2-5 MHz convex transducer by the trans-abdominal method. Ultrasound was performed in the supine position of the mother. When examining Doppler parameters, all fetuses had sinus rhythm, and the Doppler examination was performed when the fetus was not breathing. The same examiner performed all measurements.

At the beginning of the study in the non-exposed group and before the administration of betamethasone in the exposed group, ultrasound and fetal examination were performed, EFW (Estimated Fetal weight) was measured, and a general sonographic examination of the fetal heart was performed for evidence of fetal heart abnormalities. Also, the following Doppler values were measured: Umbilical Pulsatility Index (UMB PI), Middle Cerebral Artery Pulsatility Index (MCA PI), Middle Cerebral Artery Peak Systolic Velocity (MCA PSV), Pulmonary Artery Pulsatility Index (PA PI), Pulmonary Artery Resistance Index (PA RI), Pulmonary Artery Acceleration Time (PA AT), Pulmonary Artery Ejection Time (PA ET), and Pulmonary Artery AT/ET.

Doppler examination of the umbilical artery was performed in a free segment of the umbilical cord. Doppler examination of the pulmonary artery was performed in a short-axis view when the pulmonary valve and bifurcation and the right and left pulmonary branches were seen. In this study, the pulse doppler sample gate was placed in the middle of the main pulmonary artery (MPA) between the pulmonary valve and the bifurcation site. Doppler examination of the middle cerebral artery (MCA) was performed in the transverse position of the fetal head and a suitable axial section. The MCA Doppler examination was performed in the proximal third portion of the artery.

In all cases of Doppler examination, after applying the image magnification as much as possible, a sample gate of 2 mm was used; also, the angle of insonation (between sound beam and direction of blood flow) was kept below 20 degrees. The blood flow waveform was then displayed with a velocity range of 100 cm/s and a sweep speed of 200 mm/s. The shortest time interval that could be measured was 1 ms.

After obtaining the appropriate Doppler waves, each parameter was measured 3 times and their average was recorded in the patient form. The values of the pulsatility index and resistance index were measured automatically by the ultrasound machine and the values of AT and ET were measured manually as below.

AT (Acceleration time): between the foot to the top of PSV (peak systolic velocity).

**Fig. 1:**
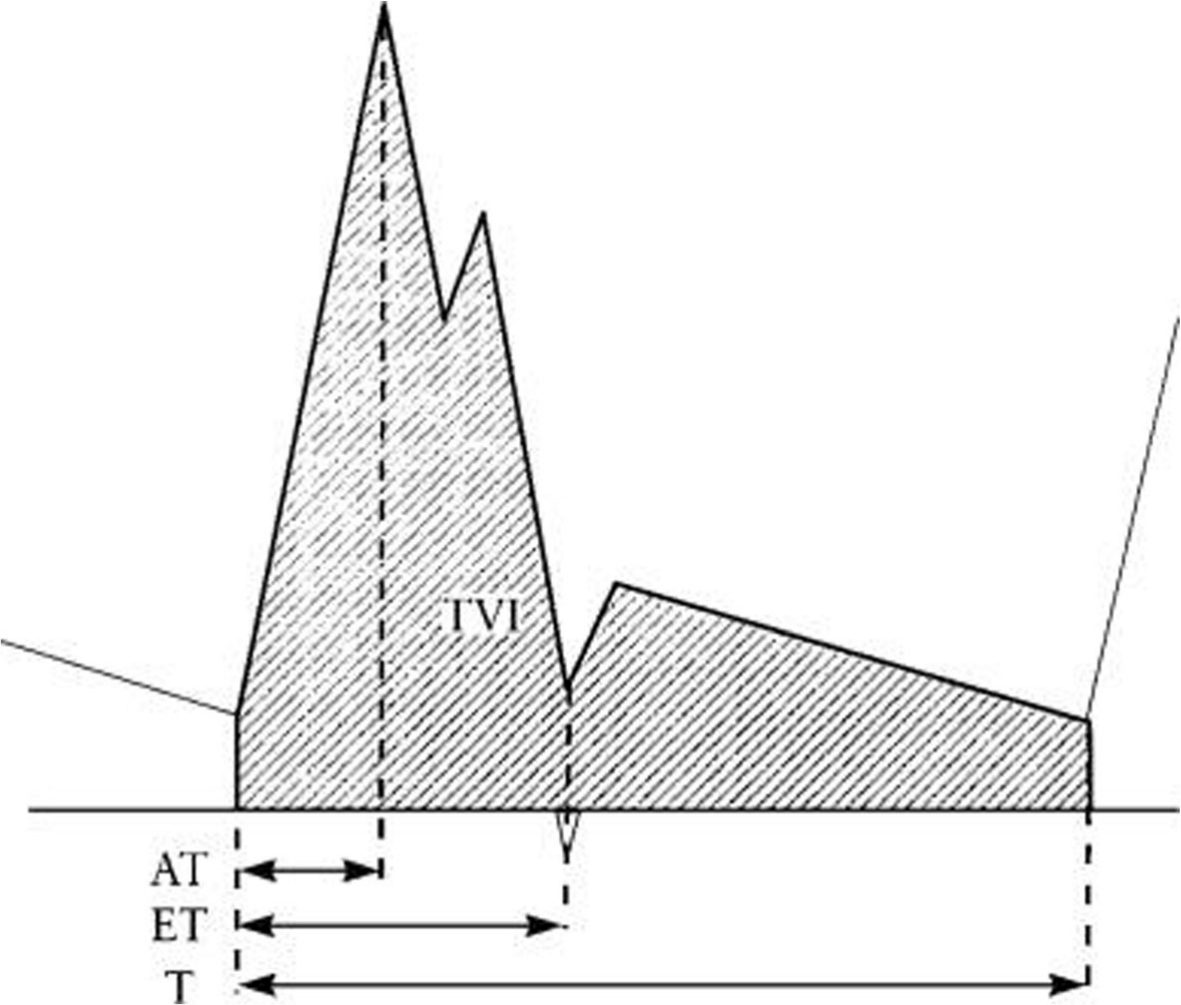
Schematic presentation of a Doppler flow velocity waveform of main pulmonary artery. AT: acceleration time; ET: ejection time; T: one heart cycle [23]

ET (Ejection time): from the beginning to the end of the ventricular systole. (Fig. 1).

In the exposure group, 24 to 48 h after receiving the corticosteroid course (two doses of 12 mg betamethasone intramuscularly 24 h apart), the patient underwent ultrasonography again and the above Doppler indices were measured again. In the non-exposed group, all Doppler indices were measured and re-recorded 48 to 72 h after the initial ultrasonographic evaluation.

Also, if the fetus was born within 7 days after the last ultrasound examination, the infant was evaluated for the occurrence of Respiratory Distress Syndrome (RDS). The diagnosis of RDS in this study was based on the presence of at least two of the following three criteria: 1. respiratory signs (tachypnea, retractions and nasal flaring) and persistent oxygen requirement for more than 24 h; 2. administration of surfactant; and 3. radiographic evidence of hyaline membrane disease.

Mean ± SD and frequency (relative frequency) were used to describe the quantitative and qualitative variables, respectively. Kolmogorov-Smirnov normality test, independent t test, paired t test, regression analysis and two way ANOVA test were used to analyze the data (non-parametric test was used when necessary). Statistical analysis was performed using SPSS. v. 22. Statistical significance was defined as *P* < 0.05.

## Results

**Fig. 2:**
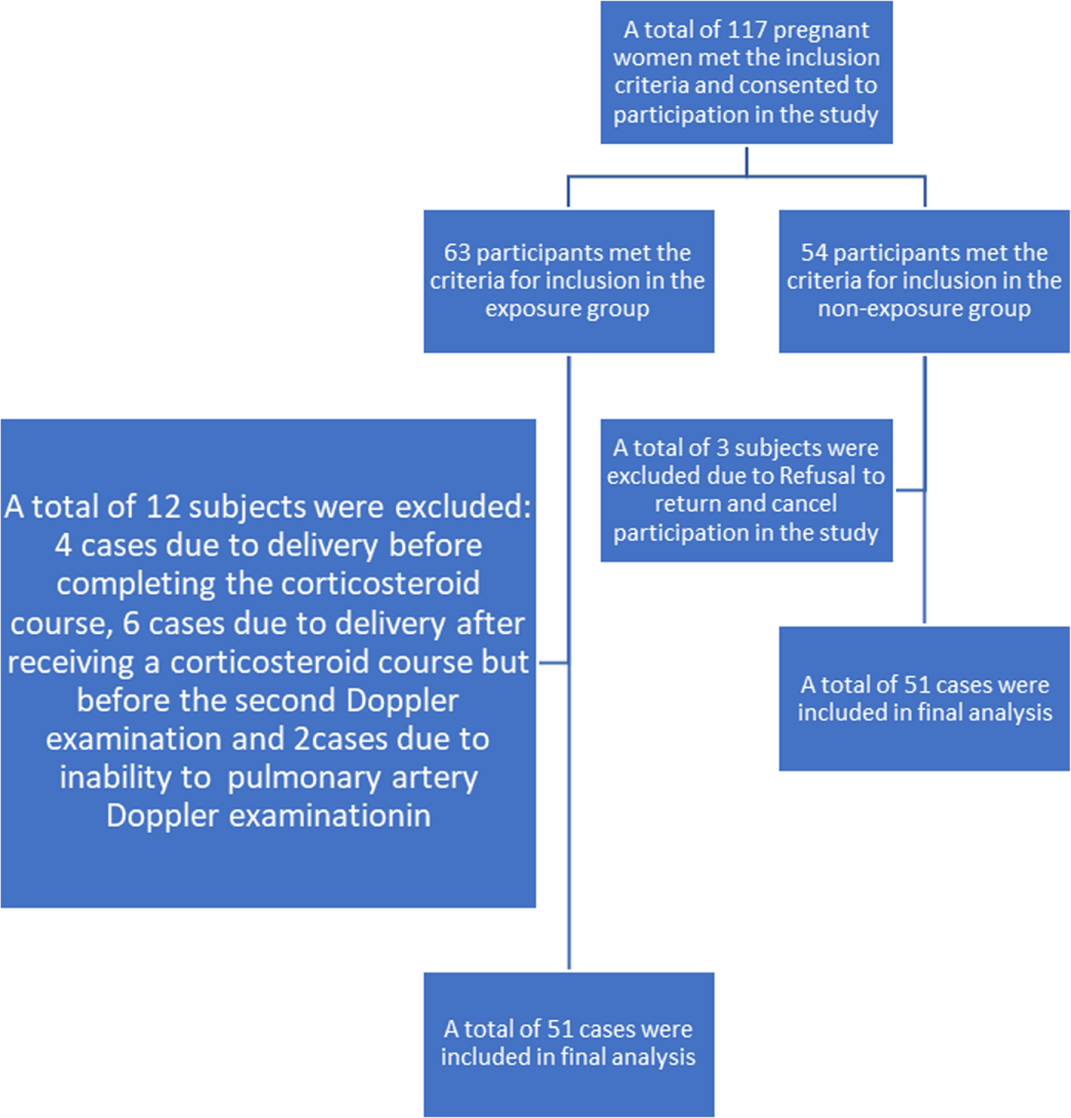
Flowchart of sample selection in the cohort of 51 exposed (betamethasone) and 51 non-exposed cases

At the beginning of the study, 63 patients in the exposure group and 54 patients in the non-exposure group were included. However, later, 12 patients in the exposure group were excluded from the study, 4 cases due to delivery before completing the corticosteroid course, 6 cases due to delivery after receiving a corticosteroid course but before the second Doppler examination, and 2 cases due to inability to examine the pulmonary artery Doppler (due to fetal position). In the non-exposed group, three patients were excluded from the study due to refusal to return for a second Doppler examination (Fig. 2).

Finally, information of 102 participants in two groups (51 participants in each group) were used in the final analysis. Demographic information about the mothers and fetuses participating in the study is shown in Table 1.

**Table 1:** Maternal and fetal features of fifty one exposed (betamethasone) and fiftyone non-exposed cases at the beginning of the study

Characteristic	Exposed *N* = 51 Mean ± SD	Nonxposed *N* = 51 Mean ± SD	*P*-value
Maternal age	28.02 ± 3.49	28.25 ± 3.14	0.19
Maternal body mass index (BMI) kg/m^2^	24.54 ± 1.46	24.40 ± 1.30	0.61
Gestational age (at entry)	30.90 ± 1.75	31.24 ± 1.65	0.31
Estimated fetal weight (EFW) (at entry)	1699.62 ± 373.6	1824.72 ± 321.0	0.073

In this study, there was no significant difference between the two groups in terms of maternal age, maternal body mass index, mean gestational age, and estimated fetal weight.

The results of measuring different Doppler parameters (first and second time of examination) in the 2 groups are shown in Table 2. Two way ANOVA analysis of these parameters is shown in Table 3.

**Table 2:** Doppler indices in the fetuses from mothers that received steroid treatment compared to the non-exposure group (did not receive steroids)

Parameter	Exposed group *N* = 51 mean ± SD	Nonexposed group *N* = 51 mean ± SD	*P*-value
Umbilical artery PI^a^			
First time evaluation	0.969 ± 0.105	0.992 ± 0.103	0.714
Second time evaluation	0.898 ± 0.079	0.991 ± 0.093	0.0001*
MCA PI			
First time evaluation	1.913 ± 0.133	1.820 ± 0.013	0.191
Second time evaluation	2.015 ± 0.239	1.881 ± 0.121	0.048*
MCA PSV^b^			
First time evaluation	35.621 ± 4.574	36.451 ± 6.096	0.256
Second time evaluation	36.052 ± 4.041	35.983 ± 4.739	0.481
Pulmonary artery PI			
First time evaluation	2.307 ± 0.361	2.072 ± 0.313	0.389
Second time evaluation	2.186 ± 0.280	2.060 ± 0.186	0.817
Pulmonary artery RI^c^			
First time evaluation	0.836 ± 0.037	0.812 ± 0.057	0.256
Second time evaluation	0.811 ± 0.041	0.829 ± 0.049	0.024
Pulmonary artery Acceleration Time (AT) ms			
First time evaluation	0.056 ± 0.013	0.057 ± 0.016	0.129
Second time evaluation	0.063 ± 0.013	0.055 ± 0.092	0.0001*
Pulmonary artery Ejection Time (ET) ms			
First time evaluation	0.265 ± 0.041	0.266 ± 0.047	0.158
Second time evaluation	0.264 ± 0.035	0.271 ± 0.083	0.180
Pulmonary artery AT/ET			
First time evaluation	0.212 ± 0.029	0.218 ± 0.048	0.211
Second time evaluation	0.229 ± 0.043	0.214 ± 0.031	0.001*

^a^pulsatility index. ^b^peak systolic velocity. ^c^resistance index

*P < 0.05 was considered statistically significant

**Table 3:** Two way ANOVA analysis of different Doppler indices in exposed (betamethasone) and nonexposed groups

Parameter	Source of variation	Grand mean ± sd	95% C.I	df	F_value	*P*_value	Partial Eta_squared	R square
UMB_PI^a^	Group	0.963 ± 0.007	(0.95–0.977)	1	18.85	< 0.001	0.08	0.14
	Time			1	6.95	0.009	0.03	
	Group*time			1171	6.88	0.009	0.03	
MCA_PI^b^	Group	1.909 ± 0.011	(1.887–1.931)	1	24.4	< 0.001	0.11	0.17
	Time			1	14.28	< 0.001	0.06	
	Group*time			1171	0.644	0.42	0.003	
MCA PSV^c^	Group	35.98 ± 0.35	(35.28–36.68)	1	3.33	0.069	0.16	0.019
	Time			1	0.07	0.888	0.001	
	Group*time			1171	0.435	0.510	0.002	
Pulmonary artery PI	Group	2.15 ± 0.21	(2.11–2.19)	1	6.49	0.066	0.19	0.11
	Time			1	2.59	0.109	0.013	
	Group*time			1171	1.75	0.187	0.009	
Pulmonary artery RI^d^	Group	0.824 ± 0.003	(0.817–0.830)	1	8.27	0.042	0.040	0.043
	Time			1	0.127	0.722	0.001	
	Group*time			1171	0.549	0.459	0.003	
Pulmonary artery acceleration time (AT)	Group	0.058 ± 0.001	(0.056–0.060)	1	5.84	0.016	0.028	0.045
	Time			1	0.931	0.336	0.005	
	Group*time			1171	2.65	0.105	0.013	
Pulmonary artery ejection time (ET)	Group	0.268 ± 0.003	(0.262–0.275)	1	0.871	0.352	0.004	0.010
	Time			1	0.484	0.487	0.002	
	Group*time			1171	0.586	0.445	0.003	
Pulmonary artery AT/ET	Group	0.217 ± 0.003	(0.211–0.222)	1	6.699	0.010	0.032	0.045
	Time			1	0.225	0.636	0.001	
	Group*time			1171	2.506	0.115	0.012	

^a^Umbilical artery pulsatility index

^b^Middle cerebral artery pulsatility index

^c^Middle cerebral artery peak systolic velocity

^d^Resistance index

At the beginning of the study, there was no statistically significant difference in the values of Doppler indices between the two groups. In the following as presented in Table 2 and Table 3, fetuses from mothers treated with corticosteroid compared to nonexposed group demonstrated significantly decreased umbilical artery PI (0.898 ± 0.079 VS. 0.991 ± 0.093, *P* < 0.05) and significantly increased MCA PI (2.015 ± 0.239 VS. 1.881 ± 0.121, *P* < 0.05), pulmonary artery AT (0.063 ± 0.013 VS. 0.055 ± 0.092, *P* < 0.05) and pulmonary artery AT/ET (0.229 ± 0.043 VS. 0.214 ± 0.031, *P* < 0.05), while all other indices were similar. Out of 51 patients in the exposure group with diagnosis of preterm labor, 25 cases were delivered within 7 days after the last measurement of Doppler parameters. During this time, no case of labor was seen in the non-exposed group. Out of 25 neonates born due to preterm labor in the exposure group, 8 cases had neonatal respiratory distress syndrome, and 17 neonates did not have symptoms of this syndrome. The mean time interval between the last Doppler examination and the onset of labor was 3.31 ± 0.86 days.

The mean gestational age in the neonates with respiratory distress syndrome was 29.04 ± 0.79 weeks and in neonates without symptoms of respiratory distress syndrome it was 31.5 ± 0.81 (*P* < 0.05). Table 4 shows the comparison and regression analysis of different fetus parameters and Doppler indices of different vessels (in the last measurement) in infants born in the desired period (1 week from the last Doppler examination) with or without RDS. Pulmonary artery AT values were significantly lower in the neonates affected by RDS (*P* < 0.05). There were no statistically significant differences in another pulmonary artery Doppler indices results between fetuses who developed RDS and those who did not (Table 4).

**Table 4:** Comparison of gestational age, neonatal weight, and Doppler indices between the fetuses that subsequently did or did not develop respiratory distress syndrome (RDS)

Parameter Mean ± SD	Neonates with RDS Number = 8	Neonates without RDS Number = 17	P	OR(95% C.I)^c^
Gestational age (weeks)	29.04 ± 0.14	31.31 ± 0.29	0.025*	0.1(0.08–0.13)
Neonatal weight (gr)	1886.6 ± 267	1950.5 ± 284	0.110	0.95(0.73–1.24)
Umbilical PI^a^	0.937 ± 0.095	0.904 ± 0.082	0.090	2.76(0.38–19.88)
MCA PI	1.853 ± 0.113	1.863 ± 0.097	0.409	0.95(087–1.04)
Pulmonary PI	2.026 ± 0.195	2.090 ± 0.176	0.420	0.87(0–1.62)
Pulmonary RI^b^	0.793 ± 0.041	0.825 ± 0.051	0.124	0.29(0.004–21.58)
Pulmonary AT (ms)	0.056 ± 0.016	0.070 ± 0.014	0.045*	0.98(0.75–0.99)
Pulmonary ET (ms)	0.242 ± 0.049	0.292 ± 0.047	0.098	1.004(0.96–1.041)
Pulmonary AT/ET	0.238 ± 0.29	0.259 ± 0.032	0.062	0.25(0.003–17.46)

^a^pulsatility index. ^b^resistance index. ^c^regression analysis was done to estimate OR(95% C.I)

*P < 0.05 was considered statistically significant

In order to evaluate different values of pulmonary Doppler indices in different gestational ages, different parameters measured at the beginning of the study were examined in three different groups of gestational age and are presented in Table 5.

- Group 1: Gestational age more than 28 weeks and less than 30 weeks
- Group 2: Gestational age 30 ≤ and less than 32
- Group 3: Gestational age ≤ 32 and less than 34 weeks (Table 5).

**Table 5:** Results of pulmonary artery Doppler indices at different gestational ages at the beginning of the study in all participants

Parameter	Group1 ^a^ *N* = 28	Group 2 ^b^ *N* = 37	Group 3 ^c^ *N* = 37	*P*-value
Number	28	37	37	
Pulmonary artery pulsatility index (PI)	2.113 ± 0.192	2.196 ± 0.124	2.070 ± 0.143	0.18
Pulmonary artery resistance index (RI)	0.823 ± 0.041	0.793 ± 0.035	0.831 ± 0.042	0.27
Pulmonary artery acceleration time (AT) (ms)	0.046 ± 0.010*	0.056 ± 0.012*	0.065 ± 0.015*	< 0.001
Pulmonary ejection time (ET) (ms)	0.257 ± 0.041	0.270 ± 0.048	0.268 ± 0.042	0.65
Pulmonary AT/ET	0.180 ± 0.023*	0.210 ± 0.018*	0.247 ± 0.042*	< 0.001

*post hoc analysis resulted in two by two difference between groups (*p*-value< 0.001)

^a^Gestational age ≥ 28 weeks and < 30 weeks

^b^Gestational age ≥ 30 weeks and < 32 weeks

^c^Gestational age ≥ 32 weeks and < 34 weeks

Distribution of fetuses by gestational age was as follows: 28–30 weeks: 28 (27.45%), 30–32 weeks: 37 (36.27%) and 32–34 weeks: 37 (36.27%). As shown in Table 5, pulmonary artery AT and pulmonary artery AT/ET values increased significantly with increasing gestational age.

## Discussion

Neonatal Respiratory Distress Syndrome is a common cause of mortality and morbidity in premature infants. The prenatal administration of corticosteroids to improve fetal pulmonary maturity is a well-known treatment used to reduce the risk of neonatal respiratory distress syndrome. The most beneficial effect of prenatal corticosteroid administration is 24 h to 7 days after completing the administration [24].

Despite the pleasing effect of steroids in improving pulmonary maturity, the mechanism of this positive effect is still unclear. Some researchers believe that the effect of corticosteroids in reducing pulmonary vascular resistance is the cause of this positive effect, but there are still different opinions on this subject.

We found significant decreases in the umbilical PI values after betamethasone administration, a finding similar to that of other reports [25, 26]. Elwany et al. demonstrated that antenatal steroids administration rose the fetal uteroplacental blood flow and significantly decreased the umbilical PI [27]. Thuring et al., Gungor et al., and Nozaki et al. obtained similar results [20, 23, 25]. Muldee et al. reviewed the articles about short term impacts of antenatal corticosteroids on fetal circulation, showing that 6 of 17 studies reported a decrease in the umbilical artery PI after steroid administration [28]. Some researchers believe that the reason for this decrease in placental resistance and umbilical PI is the increase in corticotropin-releasing hormone (CRH) after exogenous corticosteroid administration [19]. On the other hand, animal studies have attributed these changes to changes in fetal blood pressure [21, 22]. In general, more studies are needed to determine the cause of these hemodynamic changes in the fetus following corticosteroid administration.

As presented in Tables 2 and 3, among those fetuses exposed to betamethasone we found a statistically significant increase in the middle cerebral artery PI after betamethasone administration. Our results differ from those published by Elwany et al. in which the values of the middle cerebral artery decreased after steroid administration [20]. Besides, Nozaki et al. found no significant changes in the fetal middle cerebral artery velocity waveforms after corticosteroid treatment [21].

As mentioned above, some researchers [21, 22] believe that antenatal corticosteroid administration increase fetal blood pressure. According to this hypothesis, we can justify increase in middle cerebral artery resistance (MCA PI) as an autoregulation mechanism (to stabilize cerebral perfusion). However, there is currently no strong evidence to support this hypothesis. In another study by Piazze et al., a positive correlation between a middle cerebral artery (MCA) PI and lamellar bodies (LBs) count was found in preterm pregnancies [29]. The inclusion criteria in these studies have been different, which may justify these contradictory results. Another possible explanation for the different findings might be variances in treatment strategies and definitions of fetal growth retardation between the studies.

In our study, MCA peak systolic velocity (PSV), pulmonary artery PI, pulmonary artery RI and Pulmonary artery ET did not show any significant influence of betamethasone therapy, but we found significant changes in the pulmonary artery AT and Pulmonary artery AT/ET after betamethasone administration. Similar to the results of our study, Gungor et al. found no significant differences in the pulmonary artery PI and pulmonary artery RI results before and after steroid administration [25].

Lindsley et al. demonstrated that the pulmonary artery PI, pulmonary artery RI, and pulmonary artery ET did not show any significant influence of betamethasone therapy, but pulmonary artery AT significantly decreased after betamethasone administration [26]. Contrary to Lindsley et al. findings, we found a significant increase in the pulmonary artery AT after betamethasone administration. A possible explanation for this contradiction was “characteristics of participants”. In Lindsley et al.’s study, the range of gestational age and the mean of estimated fetal weight in the steroid group were significantly lower.

Unlike the authors mentioned above and us, Ustunyurt et al. described significant decreases in the pulmonary artery PI and pulmonary artery RI at 24 and 48 h after administration of the first dose of steroid [30].

As shown in Table 4, 25 fetuses from the steroid group were born within 7 days of the last Doppler examination, of which 8 had fetal respiratory distress syndrome (RDS). There was a significant difference in gestational age and pulmonary AT values compared to fetuses with RDS and those without it.

As expected, a negative correlation between gestational age and developing of respiratory distress syndrome was found. Pulmonary artery AT was significantly lower in the neonates affected by RDS (*P* < 0.05). This means that fetuses that develop RDS have higher pulmonary vascular resistance and lower pulmonary blood flow compared with fetuses that do not develop RDS. According to the results of our study, betamethasone reduces pulmonary artery resistance, which manifests as an increase in pulmonary artery AT. In addition, increased pulmonary artery AT was associated with a reduced risk of RDS in infants.

Besides, as represented in Table 5, in the evaluation of the pulmonary artery in our study, the mean pulmonary AT and pulmonary AT/ET values increased significantly with increasing gestational age. However, with increasing gestational age, no significant changes were observed in the pulmonary artery PI, pulmonary artery RI, and pulmonary artery ET.

Schenone et al. found that the pulmonary artery AT/ET and the surfactant/albumin ratio (TDX-FLMװ) were positively correlated [31]. This is similar to the results of our study and the increase in pulmonary artery AT and AT/ET after betamethasone administration. Like our study, Chaoui et al., Moety et al. and Guan et al. demonstrated that the pulmonary artery AT and pulmonary artery AT/ET increased with gestational age. Also, they found that neonates with respiratory distress syndrome had lower AT/ET levels [5, 17, 31].

In our study, only pulmonary artery AT had a negative correlation with RDS in neonates; however, we had some limitations. There were few cases of neonates with RDS (only 8) to fully examine the relationship between pulmonary artery Doppler indices and RDS.

Contrary to our report, Fuke et al. described that the pulmonary artery AT/ET ratio was persistent throughout gestation (20–39 weeks), but they found that the pulmonary artery AT/ET significantly reduced in pulmonary hypoplasia [32]. Some researchers [31, 33, 34] believed that the reduction in pulmonary arterial vascular resistance was reflected by the increase in the pulmonary artery AT and AT/ET; also, they suggested that compromised lung maturation was found to result in decreased vascularization, increased muscularization, and resistance in the peripheral vessels.

The underlying mechanism responsible for the reduction of pulmonary artery resistance after betamethasone treatment is not clear. A possible explanation for this mechanism is nitric oxide-mediated vasodilatation. Glucocorticoids induce the synthesis and secretion of placental CRH, which induces nitric oxide synthase [19, 35].

Human magnetic resonance imaging data also showed that third-trimester fetuses demonstrate remarkable pulmonary blood flow variation from one pregnancy to another, representing all-around from 4 to 30% of the combined cardiac output [36]. This evidence can justify different results of the studies.

Finally and based on our results, we can introduce fetal pulmonary artery Doppler indices as possible noninvasive method for prediction of neonatal RDS. This method had potentiality to be applied clinically, thereby avoid several invasive amniocentesis for evaluation of lung maturation.

## Conclusion

Taken together, these data recommend that maternal antenatal betamethasone administration causes significant changes in fetus blood velocity waveforms. Also, fetal main pulmonary artery Doppler indices can be measured throughout gestation and this technique is safe and reproducible for evaluation of pulmonary circulation. Although considering the disparate findings above, additional extensive studies are needed to better examine the relationship between betamethasone administration, blood velocity waveforms, Doppler indices, and neonatal outcomes.

## Data Availability

All respectable readers and researchers can request the data by directly contacting the primary author at fahim.kaveh@gmail.com

## Abbreviations

PI: Pulsatility index
AT: Acceleration time
RDS: Respiratory distress syndrome
EFW: Estimated Fetal weight
UMB PI: Umbilical Pulsatility Index
(MCA PI: Middle Cerebral Artery Pulsatility Index
MCA PSV: Middle Cerebral Artery Peak Systolic Velocity
PA PI: Pulmonary Artery Pulsatility Index
PA RI: Pulmonary Artery Resistance Index
PA AT: Pulmonary Artery Acceleration Time
PA ET: Pulmonary Artery Ejection Time
MPA: Main pulmonary artery
MCA: Middle cerebral artery
AT: Acceleration time
ET: Ejection time
LBs: Lamellar bodies
PSV: Peak systolic velocity
